# Corticotropin releasing hormone promotes inflammatory bowel disease via inducing intestinal macrophage autophagy

**DOI:** 10.1038/s41420-021-00767-8

**Published:** 2021-12-07

**Authors:** Sheng-Bing Zhao, Jia-Yi Wu, Zi-Xuan He, Yi-Hang Song, Xin Chang, Tian Xia, Xue Fang, Zhao-Shen Li, Can Xu, Shu-Ling Wang, Yu Bai

**Affiliations:** 1 Department of Gastroenterology, Changhai Hospital, Second Military Medical University/Naval Medical University, Shanghai, China; 2 Department of Gastroenterology, General Hospital of Central Theater Command, Wuhan, China

**Keywords:** Ulcerative colitis, Acute inflammation

## Abstract

Psychosocial stress is a vital factor contributing to the pathogenesis and progression of inflammatory bowel disease (IBD). The contribution of intestinal macrophage autophagy to the onset and development of IBD has been widely studied. Herein, we investigated the underlying mechanism of psychosocial stress in an IBD mouse model pertaining to macrophage autophagy. Corticotropin releasing hormone (CRH) was peripherally administrated to induce psychosocial stress. For in vivo studies, dextran sulfate sodium (DSS) was used for the creation of our IBD mouse model. For in vitro studies, lipopolysaccharide (LPS) was applied on murine bone marrow-derived macrophages (BMDMs) as a cellular IBD-related challenge. Chloroquine was applied to inhibit autophagy. We found that CRH aggravated the severity of DSS-induced IBD, increasing overall and local inflammatory reactions and infiltration. The levels of autophagy in intestinal macrophages and murine BMDMs were increased under these IBD-related inflammatory challenges and CRH further enhanced these effects. Subsequent administration of chloroquine markedly attenuated the detrimental effects of CRH on IBD severity and inflammatory reactions via inhibition of autophagy. These findings illustrate the effects of peripheral administration of CRH on DSS-induced IBD via the enhancement of intestinal macrophage autophagy, thus providing a novel understanding as well as therapeutic target for the treatment of IBD.

## Introduction

Inflammatory bowel disease (IBD), which includes ulcerative colitis (UC) and Crohn’s disease (CD), is a group of diseases characterized by the chronic and recurrent inflammation of intestinal mucosa [1, 2]. In UC, the areas of inflammation extend continuously from the rectum throughout the whole colon, and inflammation is mainly the mucosa. In contrast, inflammation in CD is characterized by patchy “skip lesions” in the distal small intestine and colon, which are transmural and accompanied by lymphoid aggregation [3, 4]. IBD pathogenesis is widely acknowledged to involve an abnormality in the intestinal defense system, comprising the mucosa, intestinal epithelial cells, and subendothelial immune cells, ultimately resulting in damage to the intestinal mucosal barrier and a disturbance in microbial homeostasis [5, 6]. Considering the potential factors responsible for the induction of intestinal defense system damage, the accumulation and activation of macrophages in the submucosa layer contributes greatly to the overinduction of various forms of inflammatory and immune responses [7, 8]. Thus, regulating macrophage-induced intestinal inflammatory and immune responses may be an effective approach in the treatment of IBD.

Psychosocial stress has been reported to be a contributing factor in the pathogenesis and progression of IBD, eliciting damage to the intestinal defense system and disturbing microbial homeostasis [9–11]. In central nervous system, the physiological response to stress lies in the triggering of hypothalamic–pituitary–adrenal axis, thus releasing corticotropin releasing hormone (CRH) for the regulation of neuroendocrine function and immune reaction [12, 13]. CRH is predominantly synthesized in the paraventricular nucleus of the hypothalamus and secreted into portal capillaries converging at the anterior lobe of the pituitary gland [14]. In addition, CRH is also widely expressed in extracranial tissues, including the endometrium, placenta, liver, stomach, and small and large intestines, but at much lower levels than that in the hypothalamus [15, 16]. Nonetheless, recent studies have revealed that peripheral CRH can also play a key role in stress-induced intestinal disturbances. In the intestine, CRH and CRH-related peptides, such as urocortin 1, 2, and 3, are produced by enterochromaffin cells, Paneth cells, colonocytes, mast cells, and mucosal macrophages. These peptides are also secreted by the myenteric plexus and submucosal plexus [17–19]. CRH in peripheral tissues can directly regulate the intestinal inflammatory and immune responses through CRH receptor 1 (CRHR1) and CRHR2 in the digestive tract, via so-called “brain–gut interactions” [20, 21]. Although peripheral CRH has been demonstrated to play an important role in the onset and development of IBD, the underlying mechanisms have not been elucidated [22, 23].

Autophagy is a vital metabolic mechanism involving the degradation of misfolded proteins and damaged cellular organelles in lysosomes for subsequent recycling [24, 25]. The formation of autophagosomes and their fusion with lysosomes to form autolysosomes is regarded as the onset of the autophagy process, and this process is termed “autophagy flux” [26]. Autophagy has been reported to be involved in the pathogenesis and progression of various diseases. In the digestive system, it was previously demonstrated that baseline autophagy is crucial for the maintenance of intestinal homeostasis, and autophagy dysfunction may play a role in the onset and aggravation of IBD [27, 28]. Conversely, abnormal or overwhelming induction of intestinal macrophage autophagy has been reported to contribute to the aggravation of IBD, ostensibly through the triggering of inflammatory responses and reactive oxidative stress [29, 30]. Hence, additional studies are necessary to explore the complex role of autophagy in IBD.

In the present study, we peripherally applied CRH to mimic psychosocial stress in a dextran sulfate sodium (DSS)-induced IBD mouse model [31]. We hypothesize that psychosocial stress contributes to the aggravation of IBD through the enhancement of intestinal macrophage autophagy. By advancing our understanding of the role of autophagy in IBD, an effective therapeutic strategy for the treatment of IBD may be revealed.

## Results

### CRH aggravated the severity of IBD

**Fig. 1:**
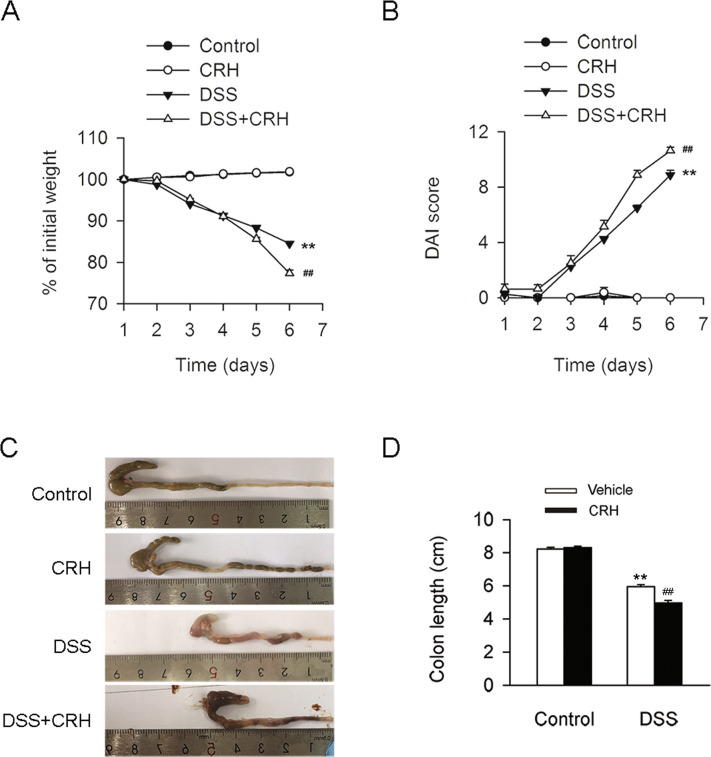
Peripheral administration of CRH contributed to the deterioration of IBD. C57BL/6 mice were administered DSS (3%) for 6 days (and a control group was provided with water only for comparison). For the CRH treatment group, CRH (or vehicle only) was intraperitoneally injected at a dose of 50 μg/kg body weight from day 1 to day 6 using saline as the vehicle. Body weight loss, stool consistency, occult or gross blood per rectum, and colon length were then recorded by two researchers who were blinded to the treatments. **A**–**D** In comparison with the DSS + vehicle group, mice in the DSS + CRH group demonstrated a significant aggravation of IBD-associated changes in body weight, DAI score, and colon length (*n* = 8 per group). **P < 0.01 vs. the control group; ^##^*P* < 0.01 vs. the DSS + vehicle group.

**Fig. 2:**
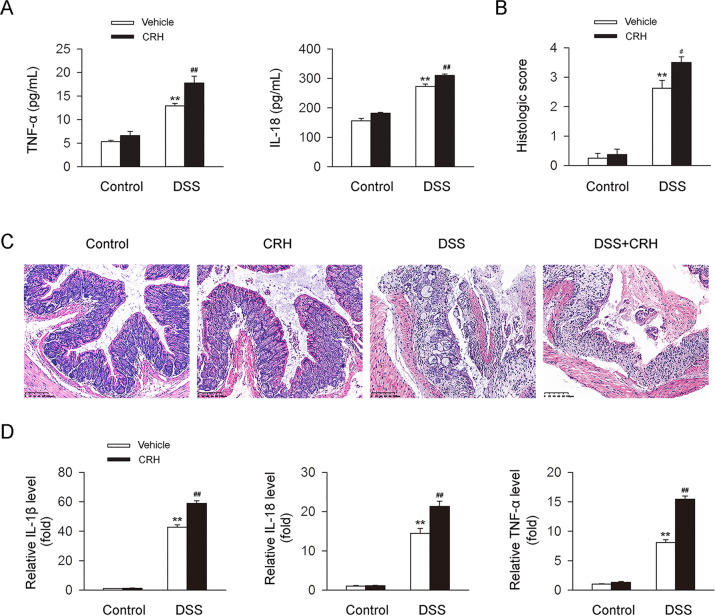
CRH contributed to the increase of inflammation in IBD mice. C57BL/6 mice were administered DSS (3%) for 6 days (and a control group was provided with water only for comparison). For the CRH treatment group, CRH (or vehicle only) was intraperitoneally injected at a dose of 50 μg/kg body weight from day 1 to day 6 using saline as the vehicle. **A** In comparison with the DSS + vehicle group, mice in the DSS + CRH group demonstrated increased levels of TNF-α and IL-18 in serum (*n* = 6 per group). **P < 0.01 vs. the control group; ^#^*P* < 0.05 vs. the DSS + vehicle group; ^##^*P* < 0.01 vs. the DSS + vehicle group. **B**, **C** The left edge of the left colon was separated and fixed, and then H&E staining was used for the detection of inflammatory infiltration (which was assessed using a histological score). In comparison with DSS-induced colitis mice, mice in the DSS + CRH group demonstrated a significant aggravation of inflammation in the left colon (*n* = 8 per group). **P < 0.01 vs. the control group; ^#^*P* < 0.05 vs. the DSS + vehicle group. **D** IL-1β, IL-18, and TNF-α mRNA levels in left colon tissues were analyzed by real-time qPCR. CRH administration also further enhanced levels of these proinflammatory factors in DSS-induced mice (*n* = 6 per group). **P < 0.01 vs. the control group; ^##^*P* < 0.01 vs. the DSS + vehicle group.

To determine the influence of psychosocial stress on IBD, peripheral administration of CRH was used to reproduce the effect of psychosocial stress in a mouse model of IBD. To generate the mouse model, C57BL/6 mice were consecutively treated with 3% DSS for 6 days. The effects of this treatment included sustained weight loss (Fig. 1A), increased DAI score (Fig. 1B), and shortened colon length (Fig. 1C, D). Subsequent administration of CRH to DSS-treated mice further aggravated these IBD symptoms (Fig. 1A–D). While levels of proinflammatory cytokines, tumor necrosis factor-α (TNF-α) and interleukin (IL)-18, were elevated in the serum of IBD mice, these levels were further elevated by CRH treatment (Fig. 2A). A histological morphology analysis of inflammatory infiltration in the left colon also revealed increased inflammation (Fig. 2B, C). Furthermore, while mRNA expression levels of proinflammatory factors in the colon, including TNF-α, IL-18, and IL-1β, were significantly elevated in IBD mice, the mRNA expression levels were further increased by CRH (Fig. 2D). Together, these results provide evidence that peripheral administration of a psychosocial stress mimic agent (CRH) could exacerbate the development of IBD.

Antalarmin and astressin2-B were applied to selectively block the CRHR1 and CRHR2 and the effect of CRH on the DSS model mice determined (Supplementary Fig. 1). While CRH administration aggravated the severity of IBD-associated body weight loss, DAI score, change in colon length, and inflammatory infiltration in the left colon, these effects were blocked by the application of antalarmin (Supplementary Fig. 1A–C, G, H). In contrast, astressin2-B administration did not significantly affect CRH-induced IBD-associated body weight loss, DAI score, change in colon length, and inflammation infiltration (Supplementary Fig. 1D–H).

In our previous study, we demonstrated that psychosocial stress was positively associated with levels of autophagy markers in intestinal samples [32]. To verify that intestinal macrophage autophagy mediated the inflammation induced by CRH in IBD, we repeated our analysis in another 12 IBD patients, and observed a similar trend (Supplementary Fig. 2). We recruited six patients with mild/moderate IBD and six patients with severe IBD. The baseline characteristics, including sex, age, disease duration, smoking history, and medications, were well balanced between the two groups; severe IBD patients showed higher CPSS scores and Mayo scores, both of which were positively related (Supplementary Fig. 2A, B). The endoscopic images, histological results (H&E staining), monocyte/macrophage infiltration (CD68 immunohistochemistry) results, and autophagy results (light chain 3, LC3 immunohistochemistry) for these patients were shown in Supplementary Fig. 2C. All above indicators were significantly increased in the colons of patients with higher reported CPSS (compared with patients with lower levels of perceived stress). In addition, the LC3-II/I ratios in intestinal biopsy specimens from patients with mild/moderate IBD were higher than those in healthy control individuals (whose basic characteristics were matched). Moreover, the LC3-II/I ratios in intestinal biopsy specimens from severe IBD patients were markedly higher. In contrast, p62/SQSTM1 marker levels were reduced, which indicated that autophagy levels were higher in IBD patients (Supplementary Fig. 2D). As shown in Supplementary Fig. 2E, autophagy levels were also positively correlated with CPSS and the modified Mayo scores.

### Peripheral administration of CRH further increased intestinal autophagy levels in IBD

**Fig. 3:**
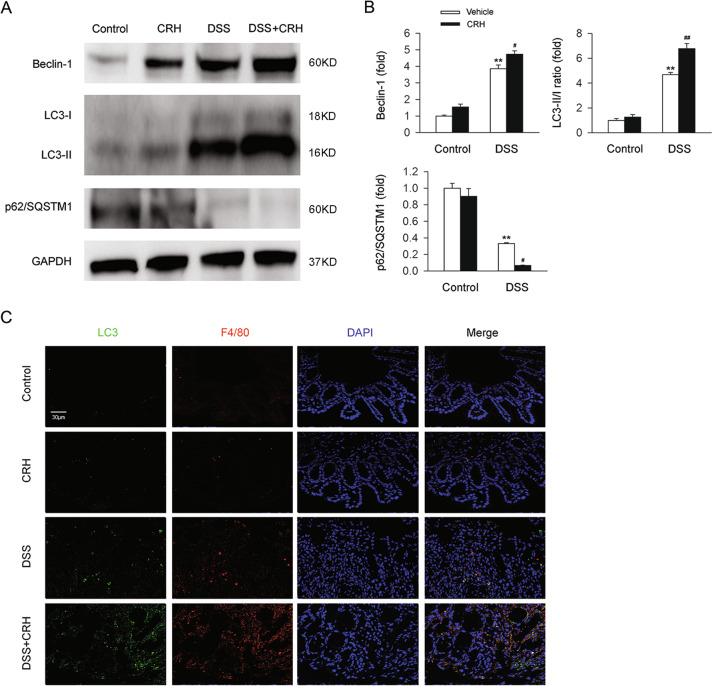
Intestinal autophagy in IBD was further enhanced by CRH. C57BL/6 mice were administered DSS (3%) for 6 days (and a control group was provided with water only for comparison). For the CRH treatment group, CRH (or vehicle only) was intraperitoneally injected at a dose of 50 μg/kg body weight from day 1 to day 6 using saline as the vehicle. **A**, **B** The levels of autophagy-related proteins, including Beclin-1, the LC3-II/I ratio, and p62/SQSTM1, were detected by western blot. In comparison with the DSS + vehicle group, mice in the DSS + CRH group exhibited significantly increased levels of Beclin-1 and the LC3-II/I ratio, but a decrease of p62 (*n* = 5 per group). **P < 0.01 vs. the control group; ^#^*P* < 0.05 vs. the DSS + vehicle group; ^##^*P* < 0.01 vs. the DSS + vehicle group. **C** The left edge of the left colon was separated and fixed and then H&E staining was used for the detection of inflammatory infiltration (which was assessed using a histological score). An increase was observed in the number of LC3 dots and the number of macrophages (which were colocalized).

High autophagy levels have been reported to play a role in the pathogenesis and progression of IBD [28, 32, 33]. In the present study, we investigated whether administration of CRH affected levels of autophagy in colonic tissues. After 6 days of treatment with 3% DSS, we observed an increase in Beclin-1 levels, an increase in the LC3-II/I ratio, and a decrease in p62/SQSTM1 levels in IBD model mice (Fig. 3A, B). These three markers of colonic autophagy were further changed after subsequent CRH administration (Fig. 3A, B). To further investigate these results, we also used immunofluorescence to evaluate the expression of autophagy markers, and the results demonstrated a similar increasing trend in the number of LC3 dots in the left colon (Fig. 3C). In addition, we labeled mouse macrophages via the F4/80 macrophage-specific marker and found that the numbers of F4/80 dots were significantly increased in the DSS + CRH group (compared to the DSS group). Moreover, the F4/80 dots were observed to colocalize with LC3 dots. Taken together, these results provide evidence that peripheral CRH further enhances intestinal autophagy in IBD, specifically colonic macrophage-associated autophagy.

### CRH administration further enhanced autophagy levels in bone marrow-derived macrophages (BMDMs) under the challenge of lipopolysaccharide (LPS)

**Fig. 4:**
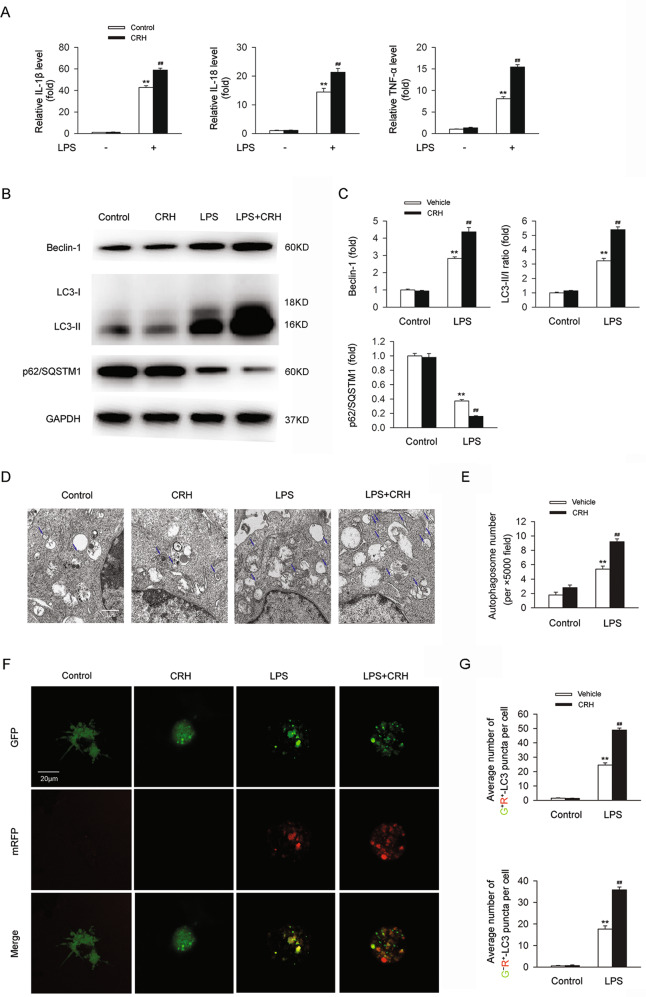
Autophagy in BMDMs challenged with LPS was aggravated by CRH. BMDMs from C57BL/6 mice were isolated and treated with/without LPS (100 ng/mL)/CRH (10^−8^ M) for 24 h. IL-1β, IL-18, and TNF-α mRNA levels were analyzed by real-time qPCR. The levels of autophagy-related proteins were analyzed in western blots. Autophagosome number was evaluated using transmission electron microscopy. Autophagy flux was assessed using the mRFP-GFP-LC3 plasmid. **A** In comparison with LPS-treated BMDMs, BMDMs treated with LPS + CRH exhibited a significant increase in the mRNA levels of proinflammatory cytokines (*n* = 6 per group). **P < 0.01 vs. the control group; ^##^*P* < 0.01 vs. the LPS + vehicle group. **B**, **C** In comparison with the LPS + vehicle group, BMDMs in the LPS + CRH group demonstrated an increase in Beclin-1, an increase in the LC3-II/I ratio, and a decrease in p62/SQSTM1 (*n* = 5 per group). **P < 0.01 vs. the control group; ^##^*P* < 0.01 vs. the LPS + vehicle group. An increase in the number of autophagosomes (**D**, **E**) (*n* = 5 per group) and an increase in autophagy flux (**F**, **G**) were also observed (*n* = 6 per group). **P < 0.01 vs. the control group; ^##^*P* < 0.01 vs. the LPS + vehicle group.

Since macrophages also play an important role in the pathogenesis and progression of IBD through activation of the innate immune system, activation of the acquired immune system, and by triggering the production of proinflammatory factors, we further explored the effects of CRH on autophagy in BMDMs under the challenge of LPS in vitro. The mRNA expression levels of IL-1β, IL-18, and TNF-α were significantly elevated in BMDMs treated with LPS, and these levels were further increased by CRH (Fig. 4A). In addition, levels of Beclin-1 and the LC3-II/I ratio were increased, and the level of p62 was decreased, in BMDMs stimulated by LPS (compared with the normal group). Moreover, administration of CRH markedly enhanced the levels of these autophagy-related indicators (Fig. 4B, C). Similar trends were observed in the numbers of autophagosomes in BMDMs (Fig. 4D, E) and in the autophagy flux (Fig. 4F, G). These results demonstrate that CRH further enhances the level of autophagy in BMDMs under the challenge of LPS.

When BMDMs were cocultured with colonic organoids in vitro, the activated macrophages negatively affected intestinal-derived cells (Supplementary Fig. 3). First, BMDMs were plated on the top membrane of transwell inserts and incubated for a minimum of 12 h with or without LPS. When a cell attachment rate of 60% was achieved, the plated BMDMs were cocultured with mouse colonic organoids. In comparison to cocultures with resting macrophages, the growth rate of mouse colonic organoids, shown by morphological changes and the number of PCNA-positive fluorescence points, was significantly decreased in cocultures with LPS-activated BMDMs (Supplementary Fig. 3A, B). Moreover, the intestinal mucosal barrier, shown by the number of E-cadherin-positive fluorescence points, was significantly decreased in the colonic organoids cocultured with LPS-activated macrophages (Supplementary Fig. 3B).

### Induction of autophagy by rapamycin aggravated CRH-induced colonic damage in an IBD mouse model

The effects of autophagy on CRH-induced intestinal damage were also examined following rapamycin induction of autophagy (Supplementary Fig. 4). IBD-associated body weight loss, DAI score, change in colon length, and inflammatory infiltration in the left colon were all significantly aggravated in the DSS + rapamycin group (in comparison with the DSS group). Furthermore, mice in the DSS + CRH group showed aggravated IBD symptoms (compared with the DSS + vehicle group), but these symptoms were not significantly modulated by rapamycin.

### Blockade of autophagy attenuated CRH-induced colonic damage in an IBD mouse model

**Fig. 5:**
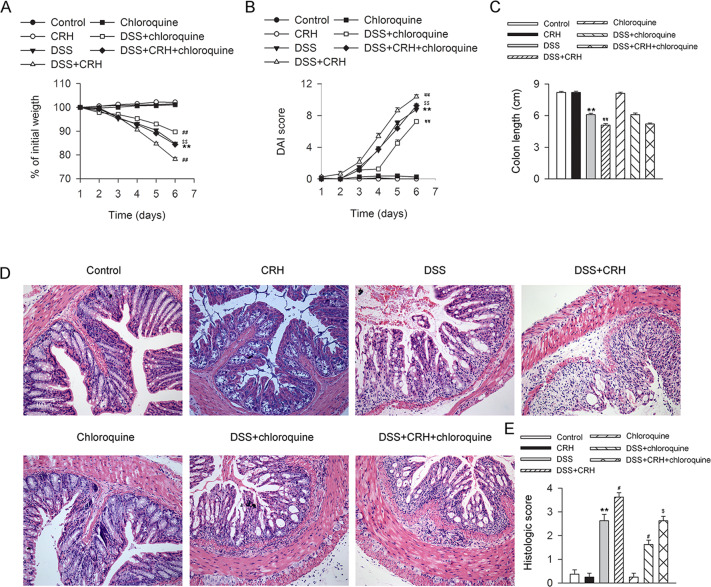
Chloroquine attenuated CRH-induced colonic damage in IBD mice. C57BL/6 mice were administered DSS (3%) for 6 days (and a control group was provided with water only for comparison). For the CRH, chloroquine, and CRH-chloroquine groups, CRH (50 μg/kg body weight) and/or chloroquine (60 mg/kg body weight) was intraperitoneally administrated from day 1 through day 6 (using saline as a vehicle). Initial body weight, DAI score, and colon length were subsequently determined by two researchers blinded to the treatment groups. In addition, inflammation in the left colon was assessed by HE staining. **A**–**C** In comparison with the DSS + vehicle group, mice in the DSS + CRH group exhibited significant increases in body weight loss, DAI scores, and colon shortening. In contrast, coadministration of chloroquine largely attenuated the aggravation effects of CRH on body weight loss and DAI Score, but no significant change in colon length (*n* = 8 per group). **P < 0.01 vs. the control group; ^##^*P* < 0.01 vs. the DSS + vehicle group; ^$$^*P* < 0.01 vs. the DSS + CRH group. **D**, **E** H&E staining was used to assess histological scores in the left colon. In comparison with the DSS + vehicle group, mice in the DSS + CRH group demonstrated an aggravation of inflammatory infiltration. In contrast, coadministration of chloroquine largely alleviated the impact of CRH (*n* = 8 per group). **P < 0.01 vs. the control group; ^#^*P* < 0.05 vs. the DSS + vehicle group; ^$^*P* < 0.05 vs. the DSS + CRH group.

**Fig. 6:**
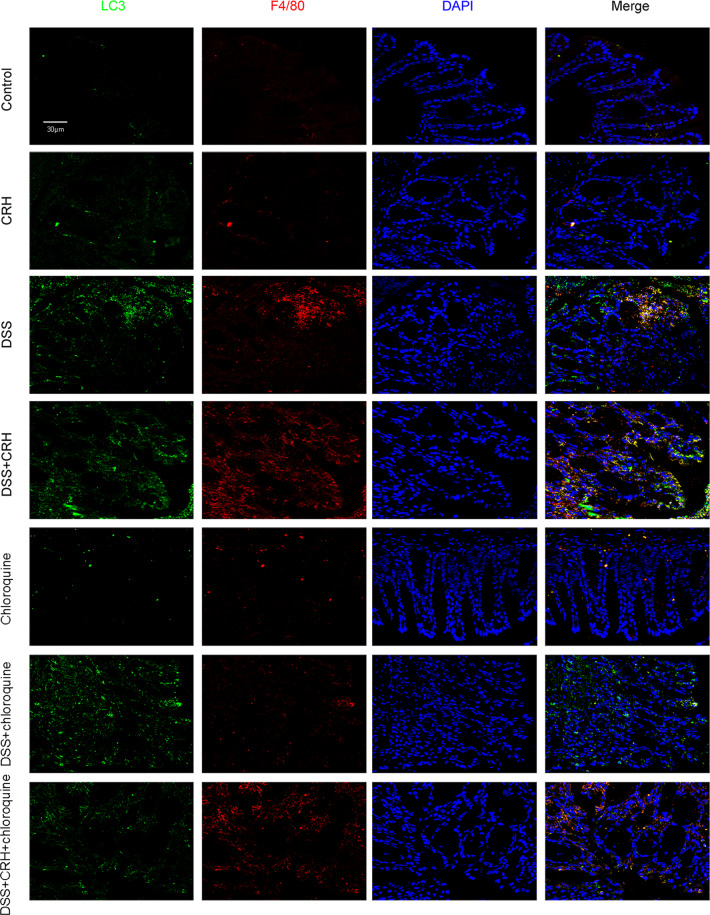
Chloroquine attenuated the increase in autophagy and number of macrophages in IBD mice. C57BL/6 mice were administered DSS (3%) for 6 days (and a control group was provided with water only for comparison). For the CRH and CRH-rapamycin groups, CRH (50 μg/kg body weight) and/or rapamycin (1.25 mg/kg body weight) was intraperitoneally injected from day 1 through day 6 using saline as a vehicle. The left edge of the colon was isolated and fixed, and immunofluorescence staining was used to assess the number of macrophages (LC3 dots) and the level of autophagy (F4/80 dots). In comparison with the DSS + vehicle group, mice in the DSS + CRH group demonstrated a significant increase in the numbers of LC3 dots and F4/80 dots. This increase was attenuated following chloroquine administration. Notably, LC3 dots colocalized obviously with the F4/80 dots.

To investigate the role of autophagy in CRH-induced intestinal damage in IBD mice, we used chloroquine, which blocks the binding of autophagosomes to lysosomes, to elicit a pharmacological blockade of the autophagy process and subsequently evaluated the severity of IBD. While peripheral administration of CRH aggravated IBD-associated body weight loss, DAI score, and change in colon length, blocking the autophagy process with chloroquine significantly alleviated IBD-associated changes in body weight loss and DAI score, but not the change in colon length (Fig. 5A–C). In addition, chloroquine suppressed inflammatory infiltration in the left colon resulting from DSS-induced colitis (Fig. 5D, E). Chloroquine also attenuated CRH-induced enhancement of autophagy in the left colon, reduced the number of macrophages, and reduced the number of LC3 dots that colocalized with F4/80 dots (Fig. 6). These data provide evidence that autophagy is required for CRH-induced colonic damage in our IBD mouse models.

The role of autophagy in CRH-induced intestinal damage in IBD mice was also verified using 3-MA, another autophagy inhibitor. While CRH significantly aggravated the severity of IBD (as assessed by IBD-associated body weight loss, DAI score, change in colon length, and inflammatory infiltration), 3-MA administration significantly attenuated the detrimental effects of peripheral administration of CRH on body weight loss and DAI score, but not change in colon length (Supplementary Fig. 5A–C). Moreover, 3-MA significantly alleviated inflammation in the DSS + CRH + 3-MA group (in comparison with the DSS + CRH group) (Supplementary Fig. 5D, E). Together, these results indicate the potential therapeutic effects of autophagy blockade in peripheral CRH-induced IBD.

### Chloroquine attenuated CRH-induced increases in inflammation and autophagy in BMDMs under the challenge of LPS

**Fig. 7:**
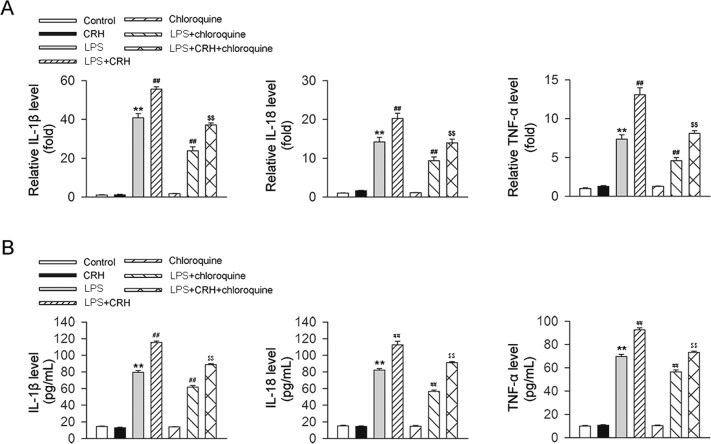
Chloroquine alleviated the CRH-induced enhancement of inflammation in BMDMs under the challenge of LPS. Murine BMDMs were isolated and subsequently stimulated with LPS (100 ng/mL), CRH (10^−8^ M), and/or chloroquine (10 μM). Real-time qPCR and ELISA were then used to assess the levels of proinflammatory factors. In comparison with the LPS + vehicle group, CRH administration further increased the levels of IL-1β, TNF-α, and IL-18 mRNA (**A**) and protein (**B**). (*n* = 6 per group.) **P < 0.01 vs. the control group; ^##^*P* < 0.01 vs. the chloroquine + vehicle group; ^$$^*P* < 0.01 vs. the chloroquine + CRH group.

**Fig. 8:**
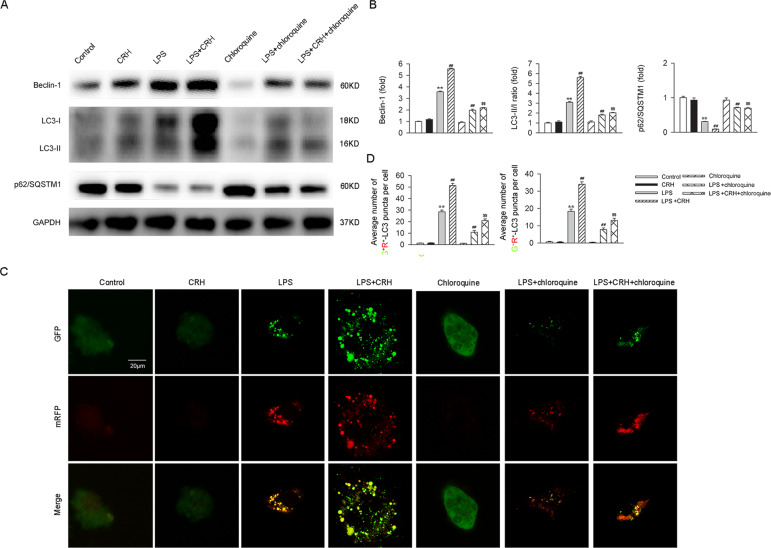
Chloroquine attenuated the CRH-induced increase in autophagy in BMDMs under the challenge of LPS. BMDMs were treated with LPS (100 ng/mL), CRH (10^−8^ M), and/or chloroquine (10 μM). Representative images of mRFP-GFP-LC3-transfected BMDMs were then assessed by immunofluorescence. **A**, **B** In comparison with the LPS + vehicle group, mice in the LPS + CRH group exhibited a significant increase in Beclin-1 and the LC3-II/I ratio, but a decrease in p62. These effects of CRH were blocked by chloroquine administration. (*n* = 5 per group). **P < 0.01 vs. the control group; ^##^*P* < 0.01 vs. the LPS + vehicle group; ^$$^*P* < 0.01 vs. the LPS + CRH group. **C** While CRH significantly elevated autophagy levels under the challenge of LPS, chloroquine administration attenuated the effects of CRH. **D** Quantitative analysis of the number of yellow autophagosomes and red autolysosomes. (*n* = 6 per group). **P < 0.01 vs. the control group; ^##^*P* < 0.01 vs. the LPS + vehicle group; ^$$^*P* < 0.01 vs. the LPS + CRH group.

Finally, we investigated whether CRH could induce an increase in inflammation via the process of autophagy in BMDMs under inflammatory loading in vitro. In murine BMDMs under the challenge of LPS, CRH administration further increased the mRNA and protein expression levels of proinflammatory cytokines, including IL-1β, TNF-α, and IL-18. However, these effects of CRH were attenuated following blockade of the autophagy process by chloroquine (Fig. 7A, B). In addition, chloroquine attenuated CRH-induced increases in autophagy-related protein levels and autophagy flux in LPS-treated BMDMs (Fig. 8). Taken together, these data provide evidence that autophagy plays a role in the CRH-induced enhancement of inflammation in BMDMs under the challenge of LPS.

## Discussion

Here, we report for the first time that intestinal macrophage autophagy is highly associated with psychosocial stress and that it promotes the pathogenesis and progression of IBD. First, we demonstrated that peripheral administration of CRH to mimic psychosocial stress induced a detrimental effect on IBD severity, and enhanced both overall and local inflammatory reactions and infiltration in a DSS-induced IBD mouse model. Autophagy, a vital metabolic mechanism, was found to be induced following the administration of CRH in macrophages both in vivo (colonic issues) and in vitro (murine BMDMs under IBD-related inflammatory stimulation). Furthermore, chloroquine application for the inhibition of overinduced autophagy alleviated IBD-associated effects in DSS-induced IBD aggravated by CRH.

Psychosocial stress is known to enhance the pathogenesis and progression of IBD by suppressing the intestinal defense system [9–11]. In general, the physiological stress response involves the triggering of the hypothalamic–pituitary–adrenal axis, thus releasing CRH for the regulation of neuroendocrine function and internal organ immune activities. However, CRH is also secreted by peripheral tissues in the digestive system and by immune cells, including Paneth cells, macrophages, and mast cells [17–19]. Elevated CRH levels damage the mucosal barrier and enhance colonic hyperpermeability, ultimately resulting in stress-related intestinal disorders [20]. Recent studies reveal that peripheral CRH plays an important role in IBD [34–37]. Indeed, the mucosal epithelial cells of colonic tissues from IBD patients have been shown to express high amounts of CRH (both protein and mRNA).

The effects of colonic CRH on IBD pathogenesis may potentially be modulated [34]. Systemic CRH deficiency reduced local inflammatory responses in colonic tissues in an experimental colitis mouse model [36]. Furthermore, peripheral CRH enhanced visceral nociception in recovery from experimental colitis models [35]. Chen et al. [37] previously reported that consecutive administration of CRH in a DSS-induced mouse model aggravated IBD. Moreover, Teitelbaum et al. revealed that peripheral administration of CRH-induced chronic stress effects and intestinal barrier dysfunction [38]. Similarly, Vanuytsel et al. reported that peripheral CRH administration reproduced the effects of psychosocial stress and triggered the onset and development of IBD [31]. CRHR1 antagonists have been proven to be effective in the alleviation of IBD via the blockade of colonic hypersensitivity induced by colonic inflammatory reactions, thus providing a novel therapeutic option for the treatment of stress-aggravated IBD [22, 36, 37, 39]. In agreement with these results, we found that CRH administration markedly aggravated the severity of IBD, enhancing body weight loss, DAI score, colon length shortening, and inflammatory infiltration. Moreover, these effects were markedly blocked by the selective CRHR1 antagonist antalarmin. In contrast, astressin2-B, a selective CRHR2 antagonist, had no significant effect on IBD. Together, these data provide evidence that macrophage-induced inflammatory reactions play an important role in CRH-mediated aggravation of IBD.

Autophagy is an important cellular physiological pathway for the degradation of long-lived proteins and damaged organelles into metabolic products and their subsequent recycling to maintain cellular homeostasis [40, 41]. The autophagy process involves the formation of autophagosomes containing sequestered cytoplasmic materials and their subsequent fusion with lysosomes to form functional autolysosomes. Although baseline autophagy was considered to be important in the maintenance of intestinal homeostasis [28], overinduction of autophagy may aggravate IBD through the induction of autophagic cell death, thus leading to a disruption in the intestinal barrier and the excessive production of proinflammatory cytokines [30, 42]. Chloroquine, a classic autophagy inhibitor, could potentially be repurposed as a therapy for the treatment of IBD, regulating cellular metabolism and modulating inflammatory activities [42–45].

In our in vivo studies, DSS was applied to mimic IBD in a mouse model. Since macrophages are one of the major cells responding to CRH-induced stress in IBD, our specific aim was to study autophagy in macrophages. To complement our in vivo studies, we conducted a series of in vitro studies using isolated macrophages. These in vitro studies provide scientific rigor and a validation for our hypotheses. BMDMs were chosen for these studies because they are widely acknowledged to be a primary macrophage and are known to be suitable for use in vitro studies. Previous studies from our lab (and others) have demonstrated that LPS can be used to mimic DSS-mediated stress in macrophages in in vitro studies [32, 46, 47]. Thus, in vitro studies involving LPS stimulation of BMDMs should provide a dependable model from which we can draw solid conclusions.

Using our in vivo and in vitro models, we demonstrate that macrophage autophagy was significantly increased in colonic tissues in vivo (after DSS-induced IBD) and in murine BMDMs in vitro (after LPS stimulation). In both cases, peripheral administration of CRH further enhanced the levels of macrophage autophagy. In addition, chloroquine inhibition of autophagy was observed in both our DSS-induced IBD mouse model and in murine BMDMs. Chloroquine and 3-MA treatments largely alleviated the detrimental effects of CRH in DSS-induced mice. However, rapamycin did not significantly aggravate the effect of CRH on DSS-induced mice. This may be explained by the presence of severe IBD symptoms in the DSS + CRH group and/or the small sample size. Taken together, these findings provide evidence of the involvement of macrophage autophagy in the detrimental effects of CRH on IBD. Moreover, they demonstrate the therapeutic effect of the blockade of macrophage autophagy in DSS-induced colitis involving peripheral CRH.

In the present study, we reveal that CRH can promote IBD by inducing intestinal macrophage autophagy. To explore specific autophagy mechanisms in IBD-related macrophages, we previously applied an autophagy-specific microarray to monitor the expression profiles of autophagy genes in our IBD mouse model [32]. We found that the levels of several vital autophagy-related genes and proteins were significantly up-regulated in the DSS + CRH group when compared to the DSS group. In addition, CRH increased the levels of Beclin-1, Atg16L1, PIK3R4, and Atg4B proteins, but decreased the levels of GAA, CTSD, and PPKAA1 proteins (in western blot analyses). In consideration of limitations in the contents and theme of our present study, we did not explore further the specific mechanisms underlying macrophage autophagy. We acknowledge that this omission may limit the longitudinal depth and clinical translation of our current work. However, we plan to further investigate these mechanisms in subsequent research.

Taken together, we have demonstrated the aggravation effect of psychosocial stress mediated by peripheral administration of CRH on DSS-induced IBD via induction of macrophage autophagy. We believe that these findings could further improve our understanding of the pathogenesis and progression of IBD, and provide a novel insight into IBD treatments. However, to ultimately develop effective therapeutic strategies for IBD that target macrophage autophagy, additional mechanistic studies are required.

## Materials and methods

### Animal care and use

C57BL/6 mice were provided by Shanghai Super-B&K Laboratory Animal Corp., Ltd. (Shanghai, China), and kept at 22 °C under a 12-h light/dark cycle with free access to water and a standard rodent diet. At the end of the experiments, the mice were anaesthetized with phenobarbital sodium (60 mg/kg, i.p.) and euthanized using cervical dislocation unless indicated otherwise. All of the procedures were approved and conducted in accordance with the guidelines of the Animal Care Committee of Navy Medical University, Shanghai, China.

### Induction of IBD mouse model

The IBD mouse model was induced in C57BL/6 mice (8–10-week-old males) with 3% DSS (mol. wt. 36,000–50,000 kDa, MP Biomedicals LLC, Santa Ana, CA, USA) dissolved in drinking water provided *ad libitum* for 6 days (day 1–day 6). For certain mice, CRH (50 μg/kg body weight, Tocris, Ellisville, MO, USA) and/or chloroquine (60 mg/kg body weight, Sigma-Aldrich, St. Louis, MO, USA) were intraperitoneally injected from day 1 through day 6 (with saline as a vehicle).

### Disease activity score and histological analysis in mice

Body weight, the presence of occult or gross blood per rectum, stool consistency, and colon length were documented by two researchers blinded to the treatment groups. A scoring system was used to assess diarrhea and the presence of occult or overt blood in the stool [48]. Changes in body weight are reported as percentage loss of baseline body weight. The ring of the rectum was harvested postmortem, fixed in 4% buffered formalin, and embedded in paraffin. For histological analysis, sections were stained with H&E according to standard protocols. Histological scoring was evaluated in a blinded protocol by a professional pathologist. Cell infiltration was scored as: 1, an increase in inflammatory cells focally located in the lamina propria; 2, an increase in inflammatory cells extending into the submucosa; and 3, a transmural extension of the infiltrate. Tissue damage was scored as: 1, discrete lymphoepithelial lesions; 2, mucosal erosions; and 3, extensive mucosal damage and/or extension through the deeper structures of the bowel wall. The two subscores (cell infiltration and tissue damage) were added to yield a combined histological colitis severity score ranging from 0 to 6.

### Cell culture and treatment

As outlined in a previous study, murine BMDMs were obtained through the incubation of bone marrow cells [49]. In brief, bone marrow obtained from femurs and tibias was flushed, cultured, and differentiated in bone marrow growth medium composed of Dulbecco’s modified Eagle’s medium (DMEM; Gibco, Grand Island, NY, USA), 10% fetal bovine serum (FBS; Gibco, Grand Island, NY, USA), 30% L929 cell-conditioned media (source of macrophage-colony stimulating factor), and penicillin/streptomycin at 37 °C in a humidified incubator with 5% CO_2_. The bone marrow growth medium was renewed every 48 h. After 7-day cultivation, fresh medium was replaced. For additional treatments, LPS (100 ng/mL, Sigma-Aldrich, St. Louis, MO, USA), CRH (10^−8^ M, Tocris, Ellisville, MO, USA), and/or chloroquine (10 μM, Sigma-Aldrich, St. Louis, MO) were applied for 12 h.

### Enzyme-linked immunosorbent assay (ELISA)

TNF-α, IL-18, and IL-1β levels in serum and colon tissues were quantified using commercial ELISA kits in accordance with the manufacturer’s instructions (R&D system, Minneapolis, MN, USA).

### Immunofluorescence staining

Colonic tissues were fixed in 4% (w/v) paraformaldehyde overnight and embedded in paraffin. Next, 5 μm sections were cut and then processed by dewaxing and rehydration. After blocking with 5% bovine serum albumin in PBS for 2 h, the sections were incubated with F4/80 antibody (1:100, Santa Cruz Biotechnology Inc., Dallas, Texas, USA) or LC3 antibody (1:200, Novus Biologicals, Littleton, CO, USA) overnight at 4 °C. The sections were then washed three times with PBS and subsequently stained using Alexa-488- or Alexa-Cy3-labeled secondary antibody (1:500, Jackson ImmunoResearch Inc., West Grove, PA, USA) for 30 min at 37 °C. After washing, the sections were mounted on slides using Vectashield mounting medium containing 4′,6-diamidino-2-phenylindole (Vector Laboratories, Burlingame, CA, USA). Antibody colocalization was then assessed using a confocal laser scanning microscope (Fluoview FV1000, Olympus, Tokyo, Japan). All experiments were performed in a double-blind manner.

### Western blotting

Total protein was isolated from murine BMDMs and colonic tissues using a standard extraction reagent supplemented with protease inhibitor (Kangchen; Shanghai, China). The concentration of extracted protein was determined using a bicinchoninic acid protein assay kit (Beyotime Institute of Biotechnology, Haimen, China). Proteins were then separated using SDS-PAGE and electro-transferred to nitrocellulose membranes as previous described [50]. Immunoblotting was performed using a Beclin-1 antibody (1:500; Cell Signaling Technology, Danvers, MA, USA), an LC3 antibody (1:500; Novus Biologicals, Littleton, CO, USA), and a p62 antibody (1:500; Cell Signaling Technology, Danvers, MA, USA). The membranes were then incubated with an IRDye800CW-conjugated secondary antibody (Rockland, Gilbertsville, PA, USA) for 1 h at 25 °C. Finally, images of the blots were obtained using an Odyssey infrared imaging system (Li-Cor Biosciences, Lincoln, NE, USA).

### Real-time polymerase chain reaction (PCR)

Total RNA was isolated from murine BMDMs and colon tissues using TRIzol (Invitrogen, Carlsbad, CA, USA). PrimeScript RT Master Mix (Takara, Otsu, Shiga, Japan) was then used for first-strand cDNA synthesis. Real-time qPCR was performed on a 7500 real-time PCR System (Applied Biosystems™) using Fast Start Universal SYBR Green Master (Roche, Basel, Switzerland). The 2^−ΔΔCT^ method was used to analyze the qPCR results with GAPDH as an internal reference. The sequences of the primers were as follows: TNF-α (sense) 5′-AAGCCTGTAGCCCACGTCGTA-3′ and (antisense) 5′-GGCACCACTAGTTGGTTGTCTTTG-3′; IL-1β (sense) 5′-CTCGTGCTGTCGGACCCCAT-3′ and (antisense) 5′-AGTGTTCGTCTCGTGTTCGGAC-3′; IL-18 (sense) 5′-CAGGCCTGACATCTTCTGCAA-3′ and (antisense) 5′-CTCCAGCATCAGGACAAAGAAAGCCG-3′; GAPDH (sense) 5′-GTATGACTCCACTCACGGCAAA-3′ and (antisense) 5′-GGTCTCGCTCCTGGAAGATG-3′.

### Transmission electron microscopy

Murine BMDMs were harvested and fixed overnight at 4 °C in 2.5% glutaraldehyde (in 0.1 M PBS), and then postfixed in 1% buffered osmium tetroxide for 2 h. BMDMs were then processed following routine procedures and examined under a transmission electron microscope (H-700; Hitachi, Tokyo, Japan).

### Autophagy flux assessment

Murine BMDMs were isolated and cultured on slides at 37 °C. When cell confluency reached 50–70%, a tandem fluorescent mRFP-GFP-LC3 plasmid was transfected. In brief, after cultivation with DMEM supplemented with 10% FBS for 24 h, murine BMDMs were incubated with plasmids for 6 h, and then cultured in fresh DMEM supplemented with 10% FBS for 36 h. Cellular autophagosomes (G^+^R^+^) and autolysosomes (G^−^R^+^) were subsequently imaged by confocal microscopy (Leica TCS SP8, Leica, Biberach, Germany). The total number of puncta (>1 µm) per cell were counted and recorded.

### Colon organoid culture and treatment

Fresh mouse and human intestinal tissues were incubated with 2 mmol/L EDTA (Gibco, USA) for 30 min at 4 °C and 5 mmol/L EDTA (Gibco, USA) for 30 min (×2) at 4 °C on a roller. The tissues were then filtered and 10 mL of 0.1% BSA was added (Solarbio, China). Next, the isolated crypt units were plated to a density of 200 crypts per well in a 40-μL droplet of Matrigel mixed with IntestiCult media. Crypt-containing Matrigel droplets were overlaid with IntestiCult complete organoid media (Stemcell Technologies, Vancouver, BC) supplemented with penicillin/streptomycin. BMDMs were then plated on the top membrane of transwell inserts for a minimum incubation of 12 h with LPS (100 ng/mL) stimulation. When a cell attachment of 60% was achieved, the BMDMs were cocultured with colonic organoids in the presence of LPS (100 ng/mL, refresh every 24 h) to investigate the effect of BMDMs on the progression of IBD in vitro.

### Statistical analysis

Data are presented as mean ± SEM. The DAI scores among groups were analyzed by two-way analysis of variance (ANOVA) followed by Bonferroni’s post hoc test for repeated measures. For the analysis of nonparametric data and continuous variables, a Kruskal–Wallis test followed by Dunn’s post hoc test and one-way ANOVA followed by Bonferroni’s post hoc test was used, respectively. A P value < 0.05 was used to assess statistical significance. All data analyses were performed using SPSS 21.0K for Windows (SPSS, Chicago, IL, USA).

## Supplementary information

Supplemental material

## Data Availability

Data underlying this article will be shared on reasonable request to the corresponding author.
