# Supplementary material for: Corticotropin releasing hormone promotes inflammatory bowel disease via inducing intestinal macrophage autophagy
Source: Cell Death Discov. 2021 Dec 7;7:377. doi: 10.1038/s41420-021-00767-8 (PMC8648763; doi:10.1038/s41420-021-00767-8)
Supplement: Supplementary file 1 — Supplemental material [file 41420_2021_767_MOESM1_ESM.pdf]

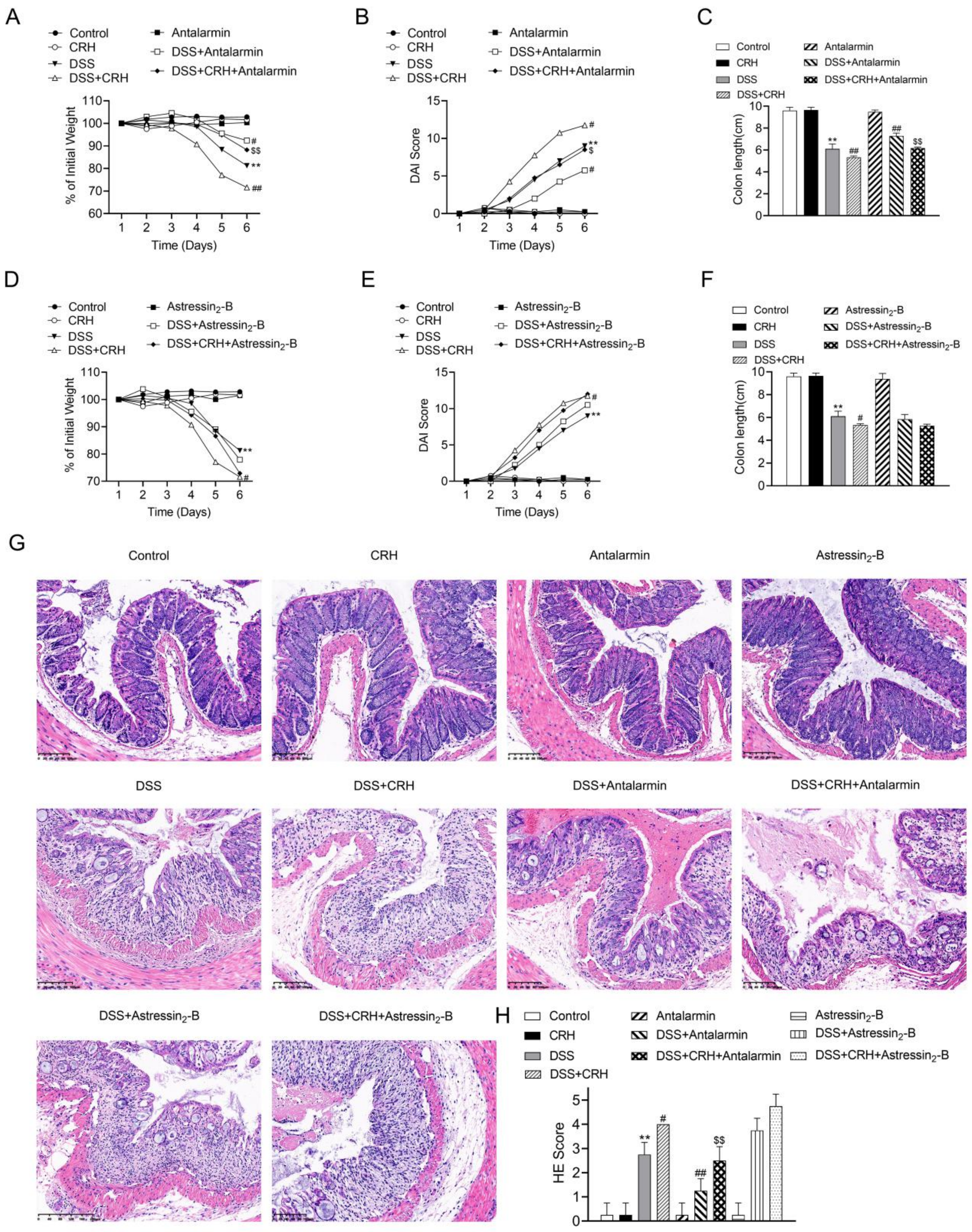

Supplementary Figure 1. Blockade of CRHR1 attenuates CRH-induced colonic damage in IBD mice.

C57BL/6 mice were administered DSS (3%) for six days (and a control group was provided with water only for comparison). Certaingroups were intraperitoneally injected with CRH (50 μg/kg body weight) and/ or the selective CRHR1 antagonist

antalarmin (20 mg/kg body weight) or the selective CRHR2 antagonist astressin$_2$-B (20 μg/kg body weight) from day 1 through day 6 (using saline as a vehicle). To demonstrate the effects of different CRHR antagonists on CRH-induced colonic damage in IBD mice, we compared antalarmin and astressin$_2$-B treatments to the control separately. (A-C) Two researchers blinded to the treatments recorded body weight, stool consistency, occult or gross blood per rectum, and colon length. IBD-associated changes in body weight, DAI score, and colon length were significantly aggravated in the DSS+CRH group (compared with the DSS+Vehicle group). The selective CRHR1 antagonist antalarmin (n=4 per group) blocked the effects of CRH on body weight loss, DAI score, and change in colon length. $^{**}P<0.01$ *vs.* the control group; $^{\#}P<0.05$ *vs.* the DSS+Vehicle group; $^{\#\#}P<0.01$ *vs.* the DSS+Vehicle group; $^{\$}P<0.05$ *vs.* the DSS+CRH group; $^{\$\$}P<0.01$ *vs.* the DSS+CRH group. (D-F) The selective CRHR2 antagonist astressin$_2$-B had no effect on the IBD-induced changes in body weight, DAI score, colon length, and colon inflammation (n=4 per group). $^{**}P<0.01$ *vs.* the control group; $^{\#}P<0.05$ *vs.* the DSS+Vehicle group. (G-H) In addition, antalarmin relieved inflammatory infiltration in the left colon resulting from DSS-induced colitis, while astressin$_2$-B did not affect colon inflammation (n=4 per group). $^{**}P<0.01$ *vs.* the control group; $^{\#}P<0.05$ *vs.* the DSS+Vehicle group; $^{\#\#}P<0.01$ *vs.* the DSS+Vehicle group; $^{\$\$}P<0.01$ *vs.* the DSS+CRH group.

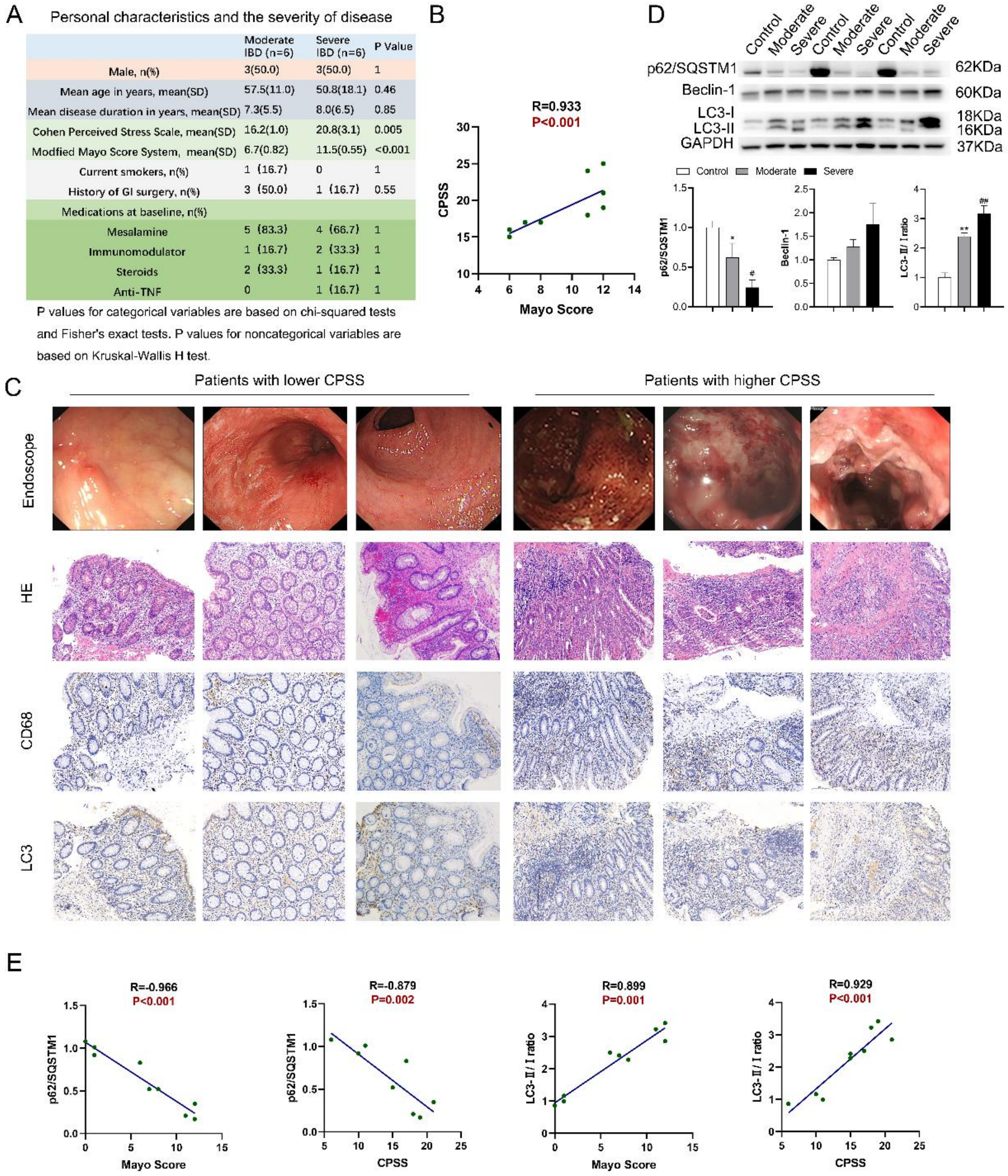

Supplementary Figure 2. Stress aggravated inflammation and increased autophagy in IBD patients.

Six mild/ moderate IBD and six severe IBD patients were enrolled. The baseline characteristics, including sex, age, disease duration, smoking history, and medications, were collated. In addition, basic characteristics and endoscopic pictures were collected during colonoscopy. The colonic biopsy tissues were fixed, and histological changes were evaluated using H&E staining. Immunohistochemical staining for LC3

was used to assess autophagy, and immunohistochemical staining for CD68 was used to detect monocytes/ macrophages. Autophagy-related proteins, including Beclin-1, LC3-II/I ratios, and p62/SQSTM1, were detected by western blotting. Correlation analysis was then used to determine correlations between autophagy-related proteins (p62/SQSTM1 and LC3-II/I ratio) and IBD severity index or CPSS scores. (A-B) The basic characteristics between the two groups were not significantly different. In comparison with the mild/ moderate IBD patient group, the severe IBD patient group demonstrated an obvious increase in CPSS (P=0.005). Moreover, an obvious relationship between CPSS and Mayo Score was observed (R=0.933, P<0.001). (C) IBD patients were divided into patients with high CPSS and patients with low CPSS. Patients with high CPSS demonstrated significantly increased inflammatory infiltration, LC3 staining, and CD68 staining in the colon (compared to patients with lower levels of perceived stress). (D) Western blot analyses for Beclin-1, the LC3-II/I ratio, and p62/SQSTM1 in biopsy specimens from healthy controls, mild/ moderate IBD patients, and severe IBD patients (n=3 per group). $^{*}P<0.05$ vs. the control group; $^{**}P<0.01$ vs. the control group; $^{\#}P<0.05$ vs. the mild and moderate IBD group; $^{\#\#}P<0.01$ vs. the mild and moderate IBD group. (E) Correlation analysis between psychosocial stress and autophagy (evaluated using p62/SQSTM1 and the LC3-II/I ratio), and correlation analysis between IBD score and autophagy (evaluated using p62/SQSTM1 and the LC3-II/I ratio) (n=3 per group).

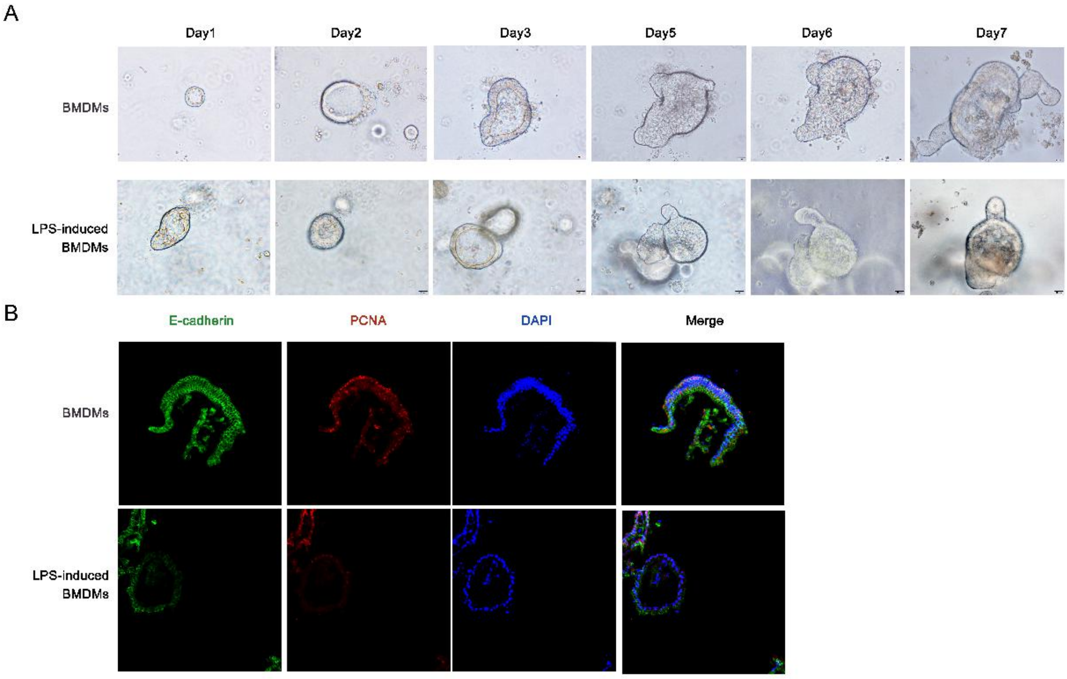

Supplementary Figure 3. LPS-induced BMDMs affected colonic organoids from mice.

Using the transwell system, we co-cultured macrophages with mouse organoids. BMDMs were plated on the top membrane of the transwell insert for a minimum of 12 h and then stimulated with LPS (100 ng/mL). At 60% cell attachment, the BMDMs were co-cultured with mouse colonic organoids to investigate the effect of BMDMs on intestinal-related cells *in vitro*. (A) Colonic organoids from mice were co-cultured with macrophages. The development of mouse organoids in the LPS-induced BMDMs group was more restricted compared with the untreated BMDMs group. (B) Immunofluorescence was used to assess E-cadherin expression (an intestinal mucosal barrier marker) and PCNA expression (a growth marker). The intestinal mucosal barrier was significantly weakened in colonic organoids co-cultured with LPS-activated macrophages.

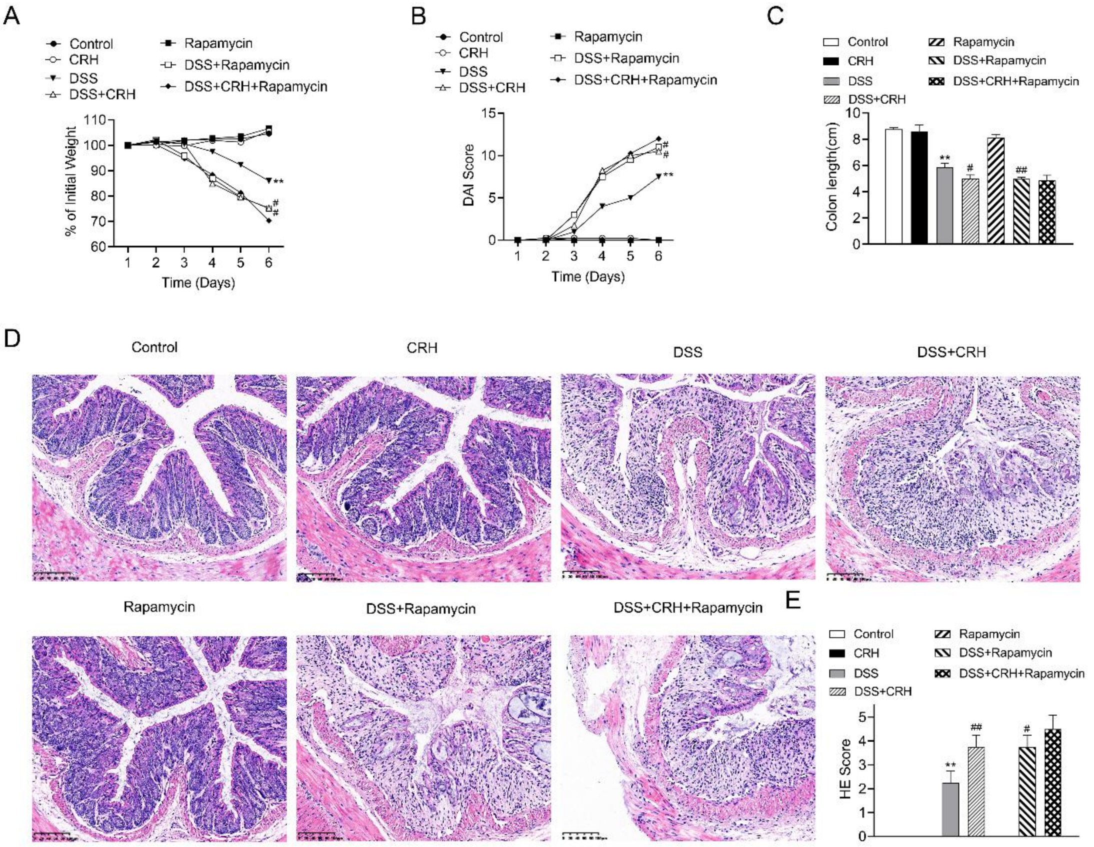

Supplementary Figure 4. Rapamycin aggravated CRH-induced colonic damage in IBD mice.

C57BL/6 mice were administered DSS (3%) for six days (and a control group was provided with water only for comparison). CRH (50 μg/kg body weight) and/ or rapamycin (1.25 mg/kg body weight) was intraperitoneally administered to certain groups from day 1 through day 6 (using saline as a vehicle). (A-C) Two investigators blinded to the treatment evaluated body weight, occult or gross blood per rectum, stool consistency, and colon length. In the DSS+CRH group, IBD-associated changes in body weight, DAI score, and colon length were significantly aggravated (compared with the DSS+Vehicle group). While these changes were not significantly further aggravated by rapamycin(n=4 per group). $^{**}P<0.01$ *vs.* the control group; $^{\#}P<0.05$ *vs.* the DSS+Vehicle group; $^{\#\#}P<0.01$ *vs.* the DSS+Vehicle group. (D-E) The left edge of

the left colon was separated and fixed and then H&E staining was used for the detection of inflammatory infiltration (which was assessed using a histological score). Mice in the DSS+CRH group demonstrated an aggravation of inflammatory infiltration in the left colon (compared with the DSS+Vehicle group), and this was not significantly affected by rapamycin (n=4 per group). $^{**}P<0.01$ *vs.* the control group; $^{\#}P<0.05$ *vs.* the DSS+Vehicle group; $^{\#\#}P<0.01$ *vs.* the DSS+Vehicle group.

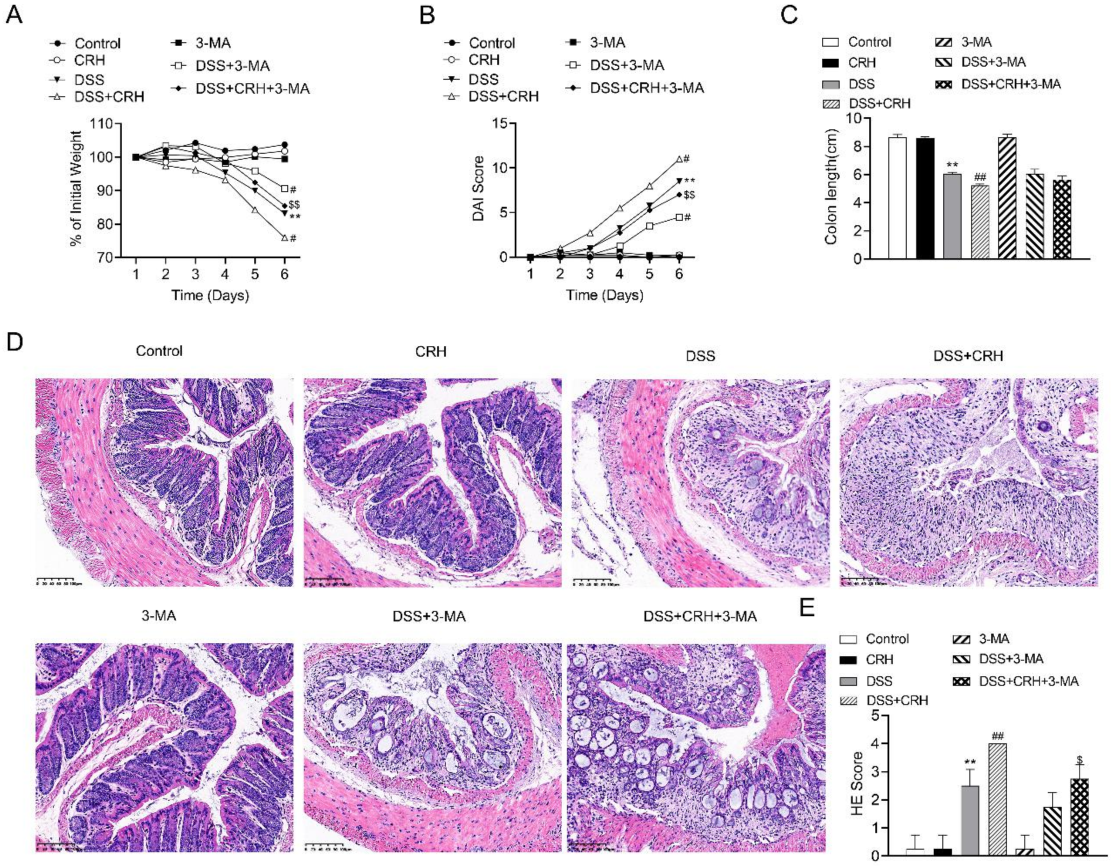

Supplementary Figure 5. 3-MA alleviated CRH-induced colonic damage in IBD mice.

C57BL/6 mice were administered DSS (3%) for six days (and a control group was provided with water only for comparison). CRH (50 μg/kg body weight) and/ or 3-MA (10 mg/kg body weight) were intraperitoneally administered to certain groups from day 1 through day 6 (using saline as a vehicle). (A-C) Two investigators blinded to the treatment groups determined body weight, the presence of occult or gross blood per rectum, stool consistency, and colon length. IBD-associated changes in body weight, DAI score, and colon length were significantly aggravated in DSS+CRH treated mice (compared with the DSS+Vehicle group). However, 3-MA application attenuated the aggravation effect of CRH on body weight loss and DAI scores, but not

on colon length (n=4 per group). $^{**}P<0.01$ *vs.* the control group; $^{\#}P<0.05$ *vs.* the DSS+Vehicle group; $^{\#\#}P<0.01$ *vs.* the DSS+Vehicle group; $^{\$\$}P<0.01$ *vs.* the DSS+CRH group. (D-E) The left edge of the left colon was separated and fixed and then H&E staining was used for the detection of inflammatory infiltration (which was assessed using a histological score). In the DSS+CRH group, mice demonstrated an aggravation of inflammatory infiltration in the left colon (compared with the DSS+Vehicle group). There was no statistical significance in the inflammatory infiltration between the DSS+3-MA group and the DSS group. However, 3-MA administration did significantly alleviate inflammation in the DSS+CRH+3-MA group (in comparison with the DSS+CRH group) (n=4 per group). $^{**}P<0.01$ *vs.* the control group; $^{\#\#}P<0.01$ *vs.* the DSS+Vehicle group; $^{\$}P<0.05$ *vs.* the DSS+CRH group.
